# Genomic insights and biosynthetic gene cluster analysis of *Streptomyces chrestomyceticus* strain B5 showing antimicrobial activities

**DOI:** 10.1128/spectrum.02688-25

**Published:** 2026-03-30

**Authors:** Aruna Kumari, Sonam Nain, Praveen Singh, Nar Singh Chauhan, Swarnendu Bag, Rakesh Sharma

**Affiliations:** 1 Genomics and Genome Biology, CSIR-Institute of Genomics and Integrative Biology, Council of Scientific and Industrial Research (CSIR) 28570 , New Delhi, India; 2 Academy of Scientific and Innovative Research (AcSIR) 550336, Ghaziabad, India; University of Melbourne, Melbourne, Australia

**Keywords:** *S. chrestomyceticus* B5, antimicrobial activity, BGCs, biosynthetic potential, transAT-PKS cluster, retention time, chromatography, antifungal compound, *C. auris*

## Abstract

*Streptomyces* bacteria are known for their different biological activities and enormous biosynthetic potential. Fungal pathogens, especially *Candida auris,* have emerged as clinically important pathogens involved in causing systemic infections and high mortality. Therefore, antimicrobial compounds effective against *C. auris* are needed to tackle infections caused by these multidrug-resistant yeast pathogens. A soil bacterium was isolated from soil in Delhi, India, exhibited broad-spectrum antimicrobial activities against various pathogenic bacteria, such as *Bacillus subtilis*, *Staphylococcus aureus,* and *Escherichia coli,* and pathogenic fungi, including *Candida albicans, Fusarium oxysporum, Aspergillus fumigatus,* and *Candida glabrata*. It was subjected to whole-genome sequencing and resulted in a high-quality draft genome with a single contig of 9.61 Mb genome size and 72.1% GC content. Phylogenetic analysis and average nucleotide identity identified that the strain belongs to *Streptomyces chrestomyceticus*. The genome annotation predicted plant growth-promoting traits and stress-response genes. Genome mining revealed non-ribosomal peptide synthetase, polyketide synthase (PKS), and hybrid biosynthetic gene clusters (BGCs) synthesizing known and unknown metabolites. Most of the encoded compounds by the BGCs were reported for antibacterial and antitumor activities, and two BGCs were identified as antifungal clusters. A trans-acyltransferase polyketide synthase (transAT-PKS) cluster was identified with 27% and 19% similarity to cycloheximide and 9-methylstreptimidone clusters, respectively. The comparison of 9-methylstreptimidone and transAT-PKS cluster revealed differences in the organization of genes in closely related strains. The crude extract of *S. chrestomyceticus* B5 possessed strong antibacterial and antifungal activities. The bioassay-guided purification and LC-MS identified an antifungal compound (streptimidone derivative or isomer, molecular mass 293.1 Da), which specifically inhibited *C. glabrata* and *C. auris*. The transAT-PKS cluster might be responsible for encoding this streptimidone derivative or isomer.

**IMPORTANCE:**

The study highlighted the biosynthetic potential of *Streptomyces chrestomyceticus* B5, which is a promising strain for producing various bioactive metabolites and is rich in biosynthetic gene clusters responsible for encoding multiple metabolites and producing an antifungal compound, a streptimidone derivative or isomer, active against drug-resistant *Candida auris*.

## INTRODUCTION

Bacterial and fungal infections continue to remain among the foremost contributing causes of global mortality, posing a significant challenge to public health around the globe. The rising antimicrobial resistance (AMR) has resulted in limited treatment options due to the ineffectiveness of present antimicrobials against pathogens. A recent study on bacterial AMR has reported 1.27 million deaths annually caused by major pathogens such as *Staphylococcus aureus, Klebsiella pneumoniae, Pseudomonas aeruginosa*, *Escherichia coli*, *Acinetobacter baumannii*, and *Streptococcus pneumoniae* (1). Fungal infections kill more than 2 million people every year, with major pathogenic genera such as *Cryptococcus*, *Candida, Aspergillus,* and *Pneumocystis*. Individuals who are immunocompromised or have underlying health conditions are more vulnerable to severe fungal infections (2). Invasive infections caused by *Candida* species affect around 1.6 million people every year and cause nearly 1 million deaths. *Candida auris,* a recently identified multidrug-resistant fungal pathogen, represents a significant global risk to human health. It spreads easily in healthcare settings, is non-susceptible to multiple antifungal drugs, can make biofilms, and survives on medical surfaces for prolonged periods. It is usually misidentified with commercially available test kits (3, 4).

A limited number of antifungal drug classes are available for treating invasive fungal infections, which is comparatively few compared with antibiotic classes. The increasing resistance to one or two major antifungals is leading to untreatable invasive fungal infections and, ultimately, death (3, 5–7). Until now, fungal infections were not considered part of AMR initiatives; however, past outbreaks with secondary infections caused by fungal pathogens in the second COVID-19 wave and the growing resistance among fungal pathogens have prompted the WHO to issue its first fungal priority pathogen list in 2022, which includes *C. auris*, *Candida albicans*, and *Aspergillus fumigatus* in the critical group, whereas *Nakaseomyces glabratus* (*Candida glabrata*) is included in the high-priority group (8).

Natural products isolated from microorganisms are known for their diverse scaffolds and complex structures and are vital in drug discovery against infections caused by pathogens (9). In particular*, Actinobacteria* and the representative genus *Streptomyces* are known to produce various bioactive molecules with different biological activities (10). They are the source of more than 70% of clinically important antibiotics (11).

Over time, efforts to discover new antimicrobials decreased due to the limitations of classical screening methods and the increasing possibility of rediscovering known molecules (12). It was thought that *Streptomyces*’ potential to produce antimicrobial compounds had been exhausted; however, genome sequencing has revealed that these bacteria hold much greater biosynthetic potential to produce new antimicrobials. However, their metabolic diversity is still untapped (13, 14). Given the extensive genomic data availability, one can effectively mine the genomes of microbes for biosynthetic gene clusters (BGCs) potentially encoding known or novel molecules using bioinformatic tools such as BAGEL, CLUSEAN, and antiSMASH (15). antiSMASH is a commonly used tool for identifying and characterizing BGCs, which can detect up to 81 biosynthetic gene cluster types (16).

*Streptomyces chrestomyceticus* produces various bioactive metabolites, including aminoglycoside antibiotic paromomycin, which binds to the small ribosomal subunit and inhibits biosynthesis of proteins (17), polycyclic tetrahydroxyxanthones, chrestoxanthones, albofungin, chlorofungin, and streptimidone (18, 19). A novel compound, chresdihydrochalcone, was recently isolated from *S. chrestomyceticus* BCC 24,770 and demonstrated bioactivity against gram-positive bacteria (19). Another novel antifungal compound with activity against *C. albicans* and *C. auris* was isolated from *S. chrestomyceticus* ADP4 (20). Therefore, mining the genomes of these *Streptomyces* species is crucial for uncovering potential biosynthetic pathways in their genomes.

In the current study, a bacterium was isolated from soil in New Delhi that was potentially active against pathogenic bacteria and fungi, and it was identified as *Streptomyces chrestomyceticus*. Genome annotation identified plant-growth-promoting traits, stress-response pathways, and DNA repair pathways. BGC analysis with other *S. chrestomyceticus* genomes predicts potential non-ribosomal peptide synthetase (NRPS) and polyketide synthase (PKS) biosynthetic pathways and several unknown BGCs with different metabolic potential. A trans-acyltransferase polyketide synthase (transAT-PKS) cluster was identified as a putative antifungal cluster that exhibits 27% and 19% similarity to cycloheximide and 9-methylstreptimidone clusters. The bioassay-guided purification resulted in the identification of an antifungal compound (streptimidone derivative or isomer) with bioactivity against *Candida* species, including drug-resistant *C. auris*.

## MATERIALS AND METHODS

### Isolation and antimicrobial activities of the strain B5

The soil sample was collected from a garden in the New Delhi area, and serial dilutions were prepared to isolate bacteria from the soil in PBS buffer. The dilutions were plated on LB agar plates and incubated at 30°C for 6 days. Following incubation, colonies with morphological characteristics resembling *Streptomyces* were chosen and further restreaked to obtain pure cultures. A distinct soil bacterial culture, B5, was screened against *E. coli* W3110 ATCC 27325*, P. aeruginosa* MTCC 424, *Bacillus subtilis* IA748, *S. aureus* MTCC 96, and *Mycobacterium smegmatis* mc^2^ 155 ATCC 700084 and fungi including *C. albicans* MTCC 227*, Saccharomyces cerevisiae* K616*, C. glabrata* MTCC 3019*, Fusarium oxysporum* MTCC 2773, and *A. fumigatus* MTCC 343 to evaluate its antimicrobial efficacy using the agar overlay method (21). A fresh culture of the strain B5 was grown on the center of an LB media plate and incubated at 30°C for 6 days. After incubation, plates were overlaid with soft agar mixed with test pathogens and then incubated overnight at 37°C (for bacterial pathogens) and 1 to 3 days at 30°C for fungal pathogens to form the zone of inhibition, and the diameter of the zone was measured in millimeters.

### Whole-genome sequencing and assembly

*S. chrestomyceticus* B5 was grown in LB broth at 30°C for 72 h. Following incubation, genomic DNA was extracted using the DNeasy UltraClean Microbial Kit (Qiagen). The genomic DNA was quantified by 0.7% agarose gel electrophoresis and further purified using AMPure XP beads (Beckman Coulter). Whole-genome sequencing was performed on the MiSeq platform (Illumina) for short reads and the MinION (Oxford Nanopore Technologies) for long reads. The *de novo* assembly was carried out using Canu 1.8 (22) with nanopore-generated long reads. Following nanopore assembly, the short reads were aligned to the long-read assembly for correcting errors using Pilon v.1.8 (23), and genome completeness was checked by CheckM (24).

### Phylogenetic analysis and whole genome-based comparison of *S. chrestomyceticus* B5

The taxonomy of the strain B5 was identified from the EzBioCloud database (https://www.ezbiocloud.net/). The 16S rRNA gene of the strain B5 was extracted using RNAmmer (25) and analyzed in the EzBioCloud database. The 16S rRNA gene sequences of the top hits were retrieved from this database and aligned with Clustal W integrated in MEGA11. The phylogenetic tree was constructed using the maximum composite likelihood model with the neighbor-joining method and 1,000 bootstrap values in MEGA11 (26). The *in silico* DNA-DNA hybridization (DDH) values were calculated using the genome-to-genome distance calculator 3.0 (https://ggdc.dsmz.de/ggdc.php) with closely related *Streptomyces* species. The comparative average nucleotide identity (ANI) values with related *Streptomyces* species were calculated using Pyani (27).

### Genome annotation and BGC analysis of *S. chrestomyceticus* B5

Three reference genomes of *S. chrestomyceticus* were downloaded from NCBI (GCF_003865135.1, GCF_017168205.1, GCF_036562025.1) for comparative genome analysis available on NCBI. Functional genome annotation was carried out using Rapid Annotation using Subsystem Technology (RAST) (28), and functionally annotated genes were assigned to different subsystems. Orthologous protein clusters of *S. chrestomyceticus* B5 and other *S. chrestomyceticus* strains were predicted using OrthoVenn3 (29). The BGCs in *S. chrestomyceticus* B5 and other *S. chrestomyceticus* genomes were predicted using antiSMASH v.7.0 (16) with default settings. The predicted biosynthetic gene cluster pathways were manually analyzed in these strains.

### Extraction and minimum inhibitory concentration of the *S. chrestomyceticus* B5 crude extract

To isolate the bioactive compounds, the *S. chrestomyceticus* B5 culture (1%) was inoculated into ISP2 media (30, 31) and incubated at 30°C for 72 h. The supernatant was collected by centrifuging at 7,500 rpm for 15 min. The cell-free supernatant was extracted with an equal volume of ethyl acetate. The organic layer was collected and evaporated to dryness in a vacuum concentrator to get the crude extract. It was resuspended in methanol and checked for antimicrobial efficacy against test microorganisms: *C. glabrata, C. albicans, A. fumigatus, S. aureus,* and *E. coli* through the agar well diffusion method. The minimum inhibitory concentration of the crude extract was also estimated against the above-mentioned pathogens. The MIC range of the crude extract was 0 μg–10 μg. The test bacterial cultures were grown at 37°C overnight and fungal pathogens at 30°C for 48–72 h. Following incubation, these cultures were diluted to an O.D. of 1 at 600 nm, and 1% of the culture was added to media tubes containing different concentrations of *S. chrestomyceticus* B5 crude extract for each test microorganism. The tubes were incubated for 18 h at 37°C, 200 rpm for test bacteria, 48 h for *Candida* species, and 72 h for *A. fumigatus* at 30°C, 200 rpm for fungi.

### Purification of antimicrobial compound from *S. chrestomyceticus* B5

Upon bioactivity assay, the crude extract was purified through silica (SiO_2_) gel column chromatography in mobile solvents, methanol:ethyl acetate (2:8), methanol, and methanol:water (8:2). Fractions, each of 2 mL, were collected, evaporated to dryness, and resuspended in 200 μL methanol, and 10 μL of each fraction was used for bioactivity screening using the agar well diffusion method.

### Reverse-phase high-performance liquid chromatography and thin-layer chromatography to purify the antimicrobial compounds

Bioactive fractions were pooled to purify on reverse-phase high-performance liquid chromatography using an Agilent C18 column. An Agilent system with a photodiode array detector was used. HPLC separation was performed with the following conditions: 0–5 min 95% A, 5% B; 5–20 min 90% A, 10% B; 20–22 min 50% A, 50% B; 22–23 min 30% A, 70% B; 23–27 min 10% A, 90% B; 27–30 min 95% A, 5% B (A: water with 0.1% formic acid, B: acetonitrile with 0.1% formic acid), with flow rate of 0.5 mL/min, and UV absorption was taken at 254 nm, and bioactive fraction was collected. After HPLC purification, the bioactive fraction was further purified using thin-layer chromatography with mobile phase acetonitrile and water (9:1). The bioactive spot was visualized under UV at 254 nm, scraped, and pooled to get the purified compound.

### Identification of bioactive compound with mass spectrometry and infrared spectroscopy

The purified bioactive antifungal compound was identified using a quadrupole time-of-flight mass spectrometer (TTOF 5600+, SCIEX) equipped with an electrospray ionization source and integrated with an Ultimate 3000 UHPLC system (Thermo Scientific). The bioactive compound was reconstituted in LC-MS grade methanol, and a 10 μL sample was used for injection. The sample was analyzed in reverse-phase mode using Acquity UPLC BEH C18 column (1.7 μm, 2.1 × 100 mm, Waters). The mobile phases used for separation were Buffer A: LC-MS grade water with ammonium acetate (10 mM) and 0.1% formic acid (vol/vol), and Buffer B: LC-MS grade acetonitrile with 0.1% formic acid (vol/vol). A 30 min gradient was applied at a flow rate of 0.2 mL/min. Ion scanning was performed in a mass range of 50 to 1,000 m/z under both positive and negative ionization modes to achieve comprehensive metabolome coverage. During each duty cycle, ions with an intensity above 100 cps in the MS1 scan and a charge of +1 were selected for MS/MS (MS2) fragmentation. A collision energy of 30 eV was applied with an energy spread of 15 eV. The infrared spectrum was recorded on an FTIR spectrometer (Thermo Fisher Scientific) over the range of 4,000 to 400 cm^−1^ using a potassium bromide pellet.

## RESULTS

### Strain B5 exhibits strong antimicrobial activities against clinically relevant pathogens

**Fig 1:**
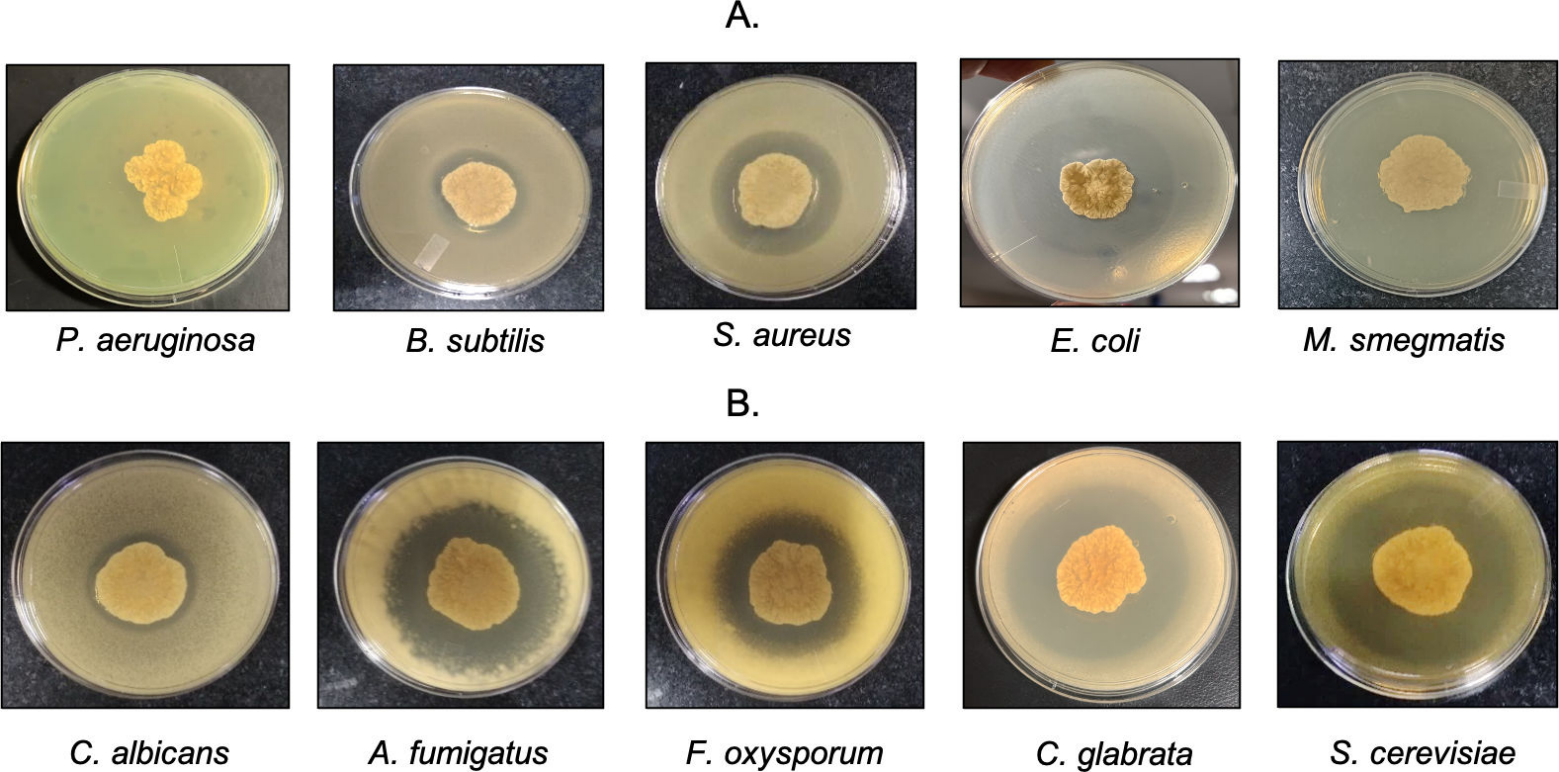
Antimicrobial potential of the B5 strain against test pathogens using the agar overlay method: (**A**) Antibacterial activity exerted by the B5 strain against gram-negative and gram-positive bacterial pathogens. (**B**) Antifungal activity displayed by the B5 strain against various fungal pathogens.

The strain B5 was filamentous in nature and formed white aerial hyphae, as observed from colony morphology. It exhibited medium inhibition toward *B. subtilis* (9 mm), followed by *S. aureus* (17 mm); however, it demonstrated strong activity against *E. coli,* with an inhibition zone of 24.2 mm. In addition, *M. smegmatis* showed complete inhibition around strain B5, forming a clear zone of inhibition, which indicates strong antibacterial activity, whereas *P. aeruginosa* showed no inhibition. (Fig. 1A). It exhibited weak antifungal activity against *C. albicans* (7 mm). It showed strong inhibition against *A. fumigatus* (20.5 mm), followed by *F. oxysporum* (21 mm), and *C. glabrata* (29 mm). The highest antifungal activity was observed against *S. cerevisiae,* with a zone of inhibition of 37.5 mm (Fig. 1B). The complete antimicrobial profile of strain B5 was summarized in Table S1.

### 16S rRNA-based phylogeny and whole genome-based comparison of the strain B5

**Fig 2:**
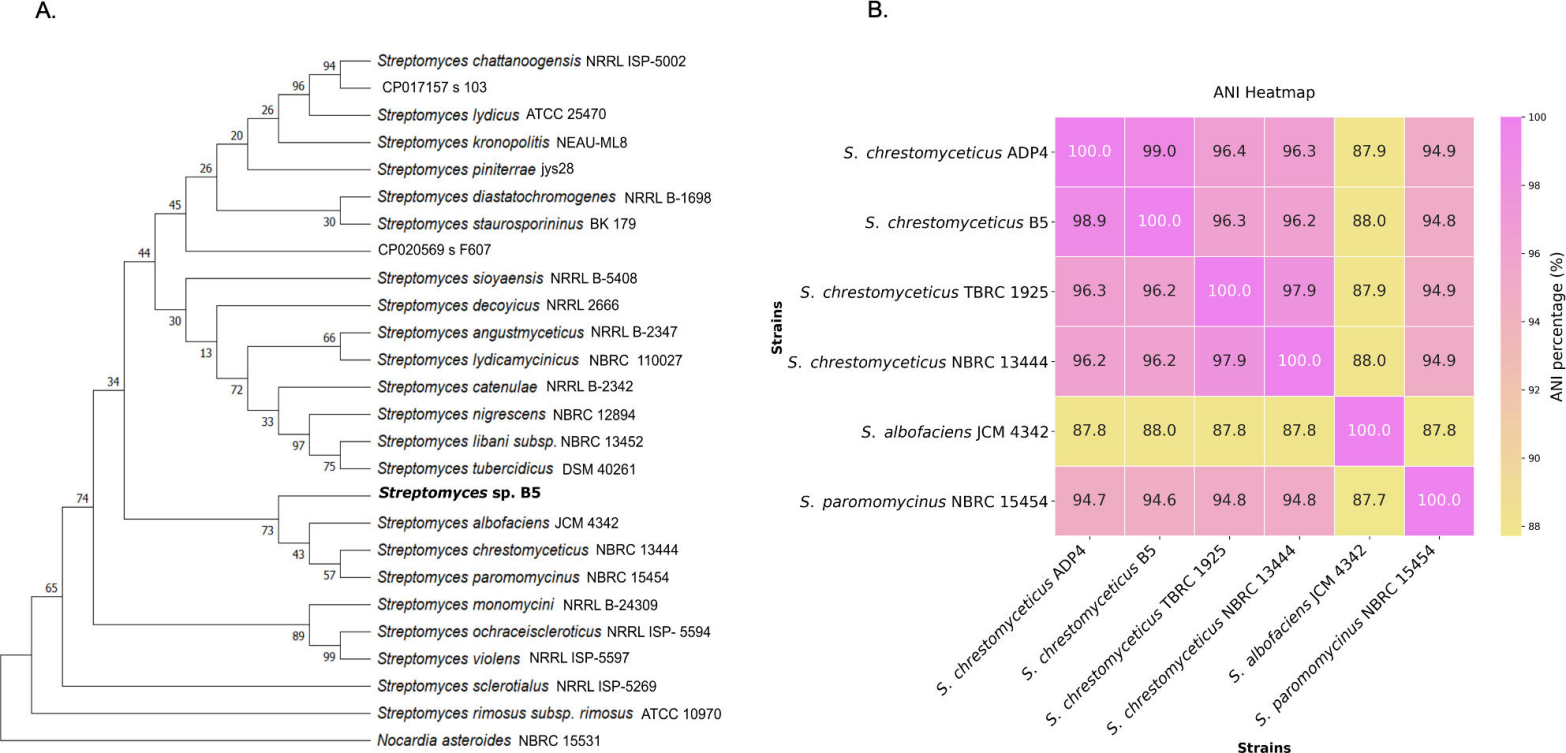
Phylogenomic analysis of B5 strain: (**A**) phylogenetic analysis based on the 16S rRNA sequence. The phylogenetic tree indicated that the B5 strain is closely related to *Streptomyces chrestomyceticus* NBRC 13444, *Streptomyces paromomycinus* NBRC 15454, and *Streptomyces albofaciens* JCM 4342. Phylogenetic analysis was done by the maximum likelihood model using the neighbor-joining method in MEGA11 with 1,000 bootstraps. *Nocardia asteroides* NBRC 15531 was considered the outgroup for the phylogenetic tree. (**B**) Average nucleotide identity heatmap of *S. chrestomyceticus* B5 with closely related *Streptomyces* species. ANI values suggested that *S. chrestomyceticus* B5 shares high genome similarity with *S. chrestomyceticus* ADP4 compared to other strains.

Based on the 16S rRNA sequence, strain B5 shared 99.93% sequence similarity with *S. chrestomyceticus* NBRC 13444 and *Streptomyces paromomycinus* NBRC 15454, and 99.52% with *Streptomyces albofaciens* JCM 4342. In the phylogenetic tree, strain B5 was grouped into a clade with *S. chrestomyceticus* NBRC 13444, *S. albofaciens* JCM 4342, and *S. paromomycinus* NBRC 15454 (Fig. 2A).

Further whole genome-based comparisons, like ANI and DDH, suggested the strain B5 belongs to *S. chrestomyceticus,* as it shared more genomic similarity with *S. chrestomyceticus* ADP4, with 98.9% ANI and 91.40% DDH (Fig. 2B; Table S2) and more than 95% ANI and 69% DDH values with *S. chrestomyceticus* NBRC 13444 and *S. chrestomyceticus* TBRC 1925 strains, respectively.

The strain B5 also formed a clade with *S. paromomycinus* NBRC 15454 and *S. albofaciens* JCM 4342 in the phylogenetic tree; however, the ANI values with these species were 94.7% and 87.8%, and the DDH values were 59.9% and 44.4%, respectively, which are less than the cutoff values to define a species (Fig. 2B; Table S2).

Therefore, based on the high ANI and DDH values of the strain B5, with *S. chrestomyceticus* strains, it was designated as *S. chrestomyceticus* B5. Three reference genomes of *S. chrestomyceticus* (NBRC 13444, TBRC 1925, and ADP4) were chosen for comparative study based on their genomic similarity.

### General genomic characteristics of *S. chrestomyceticus* B5

The genome of *S. chrestomyceticus* B5 was assembled into a single contig, with a genome size of 9.61 Mb and 72.1% GC content. The number of coding sequences was 8,918, with 86 RNA and 98.75% genome completeness (Table S3).

### Genome annotation revealed plant growth-promoting (PGP) and stress-response genes in *S. chrestomyceticus* B5

**Fig 3:**
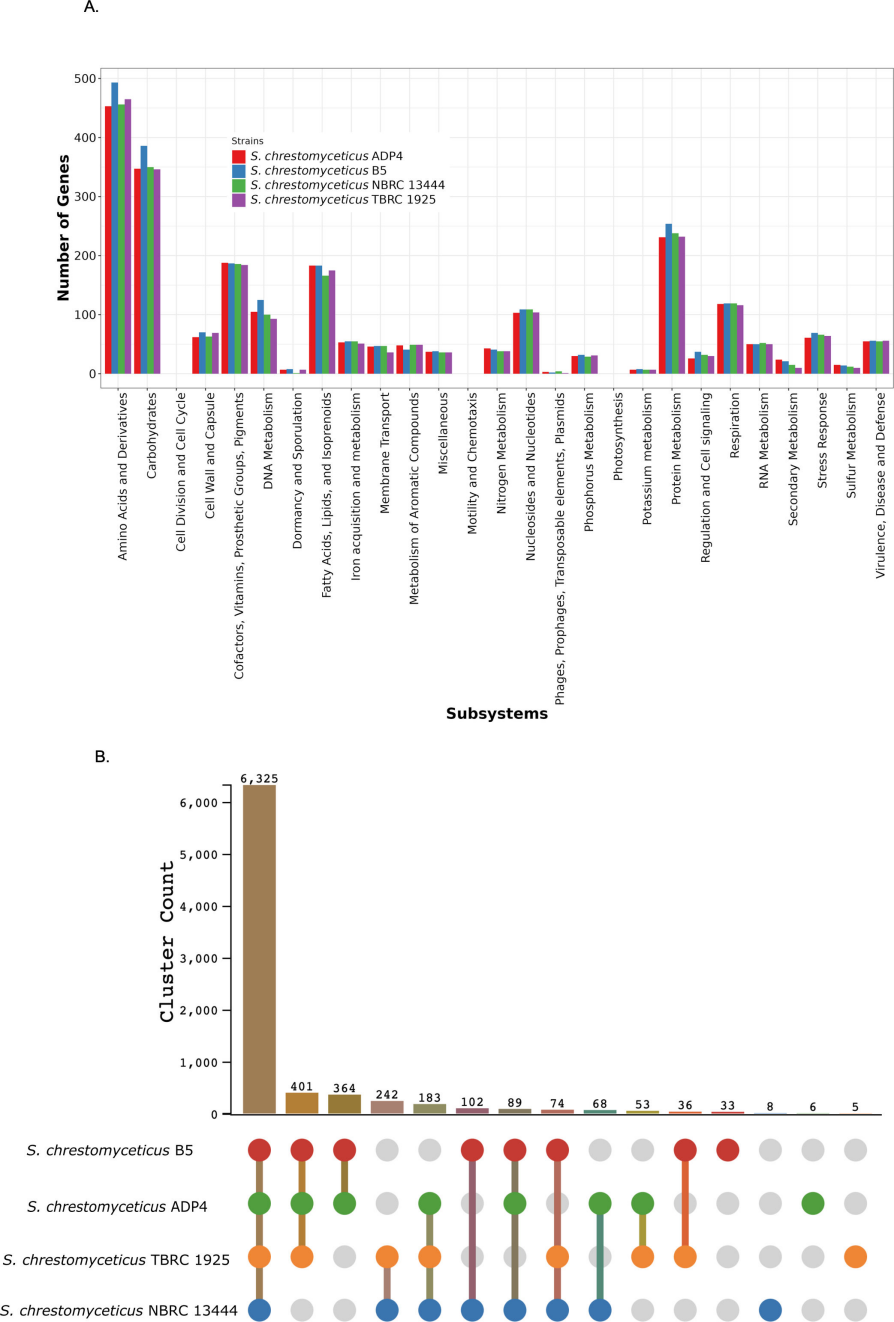
Functional annotation of *S. chrestomyceticus* strains: (**A**) RAST subsystem classification in *S. chrestomyceticus* strains. The strains contain more genes in amino acid and carbohydrate metabolism, and *S. chrestomyceticus* B5 contains more gene copies in the stress response category. (**B**) Distribution of orthologous clusters and strain-specific clusters in *S. chrestomyceticus* strains.

The comparative genome annotation analysis suggested *S. chrestomyceticus* strains had a high number of metabolism-related genes, with high-abundance genes for amino acid and derivatives, carbohydrate metabolism, followed by protein metabolism, fatty acids and lipids, cofactors and vitamin biosynthesis, and DNA metabolism, as compared to the other three *S*. *chrestomyceticus* strains (Fig. 3A). From the genome annotation analysis, it was predicted that *S. chrestomyceticus* B5 possesses PGP traits; it contains genes for siderophore production (*desABCD*), auxin biosynthesis, ammonia assimilation (*glnE, glnD, amt*), nitrate and nitrite ammonification, denitrification (*narG, narH, narJ, narI*), and phosphate-releasing genes such as exopolyphosphatase (*ppx*) and polyphosphate kinase (*ppk*). While many *Streptomyces* species have been reported for plant growth-promoting properties, such properties have not been described in *S. chrestomyceticus* strains. Therefore, the presence of these traits in *S. chrestomyceticus* B5 suggests a symbiotic relationship with plants and may be ecologically relevant. *S. chrestomyceticus* B5 also contained more gene copies for stress response as compared to the other three *S*. *chrestomyceticus* strains. It contains oxidative stress genes (*nsrR, ahpC, soxR*), osmotic stress genes (*betB, opuD, opuA*), and genes for heavy metal resistance, including cobalt-zinc-cadmium resistance (Transcriptional regulator, MerR family), copper homeostasis (*copC, copD, clfA*), and mercuric reductase (PF00070). Besides, *S. chrestomyceticus* B5 also possesses genes for DNA repair pathways such as non-homologous end joining, UvrABC system, base excision, and RecBCD pathway, containing genes for ATP-dependent DNA helicase SCO5183 and SCO5184. The *S. chrestomyceticus* B5 also possesses CRISPR-associated cas genes (*cas1, cas2*, *cas3, cse1, cas5e*).

### Comparative protein cluster analysis of *S. chrestomyceticus* strains

Comparative protein cluster analysis revealed that 6,325 clusters were shared by four *S*. *chrestomyceticus* strains, associated with various cellular and metabolic processes, which indicates a conserved core genome in these strains. *S. chrestomyceticus* B5 contains 7,424 clusters, which is comparable to *S. chrestomyceticus* ADP4 (7,489) and *S. chrestomyceticus* TBRC 1925 (7,319), but higher than *S. chrestomyceticus* NBRC 13444 (7,091) (Table S4). Beyond the core clusters, several groups of shared clusters highlighted functional differences among strains. *S. chrestomyceticus* B5, ADP4, and TBRC 1925 share 401 clusters, responsible for RNA and nitrogen compound metabolism, DNA replication and repair, and amino acid transport, suggesting similar stress response and nutrient processing in these strains. Additionally, *S. chrestomyceticus* B5 and ADP4, and *S. chrestomyceticus* B5 and NBRC 13444 share clusters responsible for ion and phosphorus transport and organic acid metabolism, thus suggesting a role for environmental adaptation in these strains. The presence of strain-specific clusters provides further insight into genomic differences. *S. chrestomyceticus* B5 possesses 33 clusters associated with nitrogen and organic acid metabolism and transposition, suggesting accessory functions that are not present in the other three strains. These results indicate that these strains share a conserved core genome, while B5 contains accessory genes which may contribute to strain-level variation (Fig. 3B).

### BGC analysis reveals strong biosynthetic potential of *S. chrestomyceticus* strains

**Fig 4:**
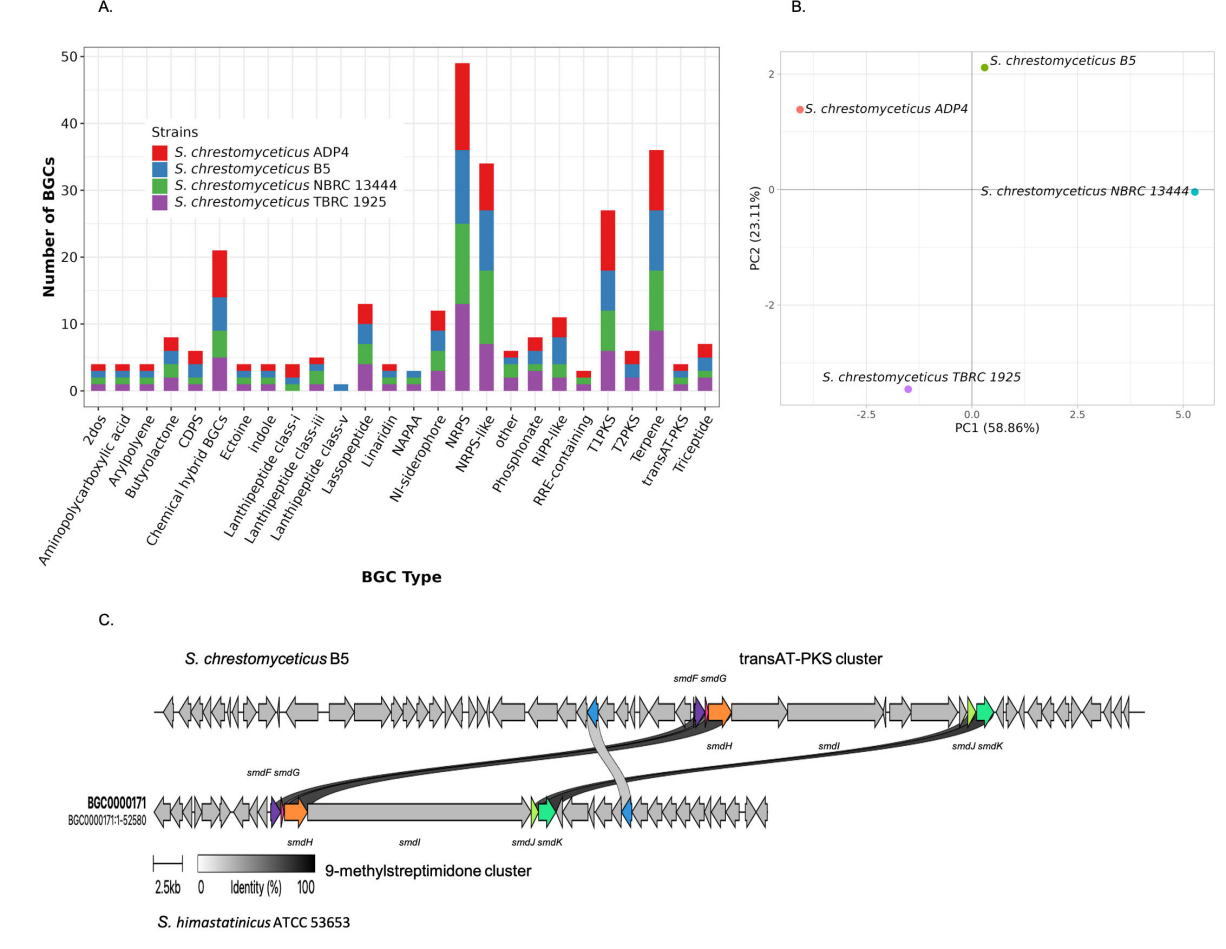
Biosynthetic diversity in *S. chrestomyceticus* strains and identification of putative antifungal cluster: (**A**) BGC classes or types predicted in *S. chrestomyceticus* strains. A few BGCs were unique to each strain. (**B**) Principal component analysis of BGC types in *S. chrestomyceticus* strains based on Euclidean distance. (**C**) Gene synteny between the BGC of 9-methylstreptidmidone from *Streptomyces himastatinicus* ATCC 53653 and the transAT-PKS cluster with putative *smd* genes in *S. chrestomyceticus* B5.

The antimicrobial activities of *S. chrestomyceticus* B5 suggested a wide antimicrobial potential toward bacteria and fungi. Therefore, we investigated the BGCs present in this strain, which could be responsible for the synthesis of various metabolites with different bioactivities. The analysis predicted 48 BGC regions with 72 BGCs in *S. chrestomyceticus* B5, with many pathways encoding metabolites with putative antibacterial, antitumor, and a few clusters with putative antifungal bioactivities. *S. chrestomyceticus* NBRC 13444 possesses 69 BGCs, *S. chrestomyceticus* TBRC 1925 has 71 BGCs, and *S. chrestomyceticus* ADP4 contains 76 BGCs. *S. chrestomyceticus* B5 contains 25 BGC classes or types, and reference strains possess 24 BGC types, with predominant BGC types including NRPS, NRPS-like (NRPS-like fragment), terpene, chemical hybrid BGCs, and PKS, and rare BGC types, such as lassopeptide, transAT-PKS, triceptide, CDPS, arylpolyene, phosphonates, linaridin, indole, and 2-deoxy-streptamine aminoglycoside (Fig. 4A), which possibly encode unknown or novel metabolites.

Several BGC types were unique to individual strains, which suggests that these strains may possess varying biosynthetic potential. *S. chrestomyceticus* B5 specifically contains the lanthipeptide-class-V BGC type, while reference strains do not. The PCA plot based on BGC types also suggested biosynthetic diversity in these strains (Fig. 4B).

### Putative secondary metabolites predicted in *S. chrestomyceticus* strains

Many of the BGCs present in these strains have high similarity to their corresponding known clusters, which suggests the possibility of encoding identical or similar metabolites. The conserved BGCs for known metabolites identified in these strains were desferrioxamine B, hopene, griseobactin, geosmin, ectoine, terpenes, and isorenieratene. The presence of these metabolites is characteristic of *Streptomyces,* as they are involved in the adaptation of these microbes in soil. The other common BGCs present in these strains possibly encode known molecules with 75 to 100% similarity to antipain, thiazostatin/watasemycin, sapB, purincyclamide, paromomycin, rimomycin, cyclothiazomycin C, and tyrobetaine clusters. Many of these BGCs are reported for bioactivity against bacteria, including *B. subtilis* and *S. aureus* (32, 33). The possible bioactivity of *S. chrestomyceticus* B5 against these bacteria could be due to any of these BGC clusters. A biosynthetic pathway shows similarity to the paromomycin cluster, which is reported for activity against gram-negative bacterial pathogens (34). *S. chrestomyceticus* B5 strongly inhibited *E. coli* that could be due to either the paromomycin pathway or some other unknown biosynthetic pathway with low similarity to known clusters.

Half of the BGCs identified in these strains share low similarity with the known clusters (less than 30% similarity), indicating the potential of these strains for novel BGCs, some of which might be involved in encoding novel or new secondary metabolites.

### Different biosynthetic capacity of *S. chrestomyceticus* strains

Few of the BGCs in these strains were encoding different metabolites specific to each strain, for example, BGC cluster for bafilomycin B1 (55%), marinoterpin A/B (12%), pristinin (23%), and hygrosin A/B (19%) were specific to *S. chrestomyceticus* B5 only (Table S5). Likewise, in reference strains, *S. chrestomyceticus* NBRC 13444 contains known BGCs for ebelactone A/B, alkylresorcinol, and cattlecin clusters, and unknown BGCs for acyldepsipeptide 1, dudomycin, and olimycin A/B clusters, which might produce new compounds (Table S6). *S. chrestomyceticus* TBRC 15454 contains BGCs for quinolidomicin A, phosphinothricin-triceptide, formicamycins A–M, chlorothricin/deschlorothricin clusters (Table S7). Although *S. chrestomyceticus* ADP4 shared high genomic similarity with *S. chrestomyceticus* B5, it also possesses specific BGCs for lysolipin I catenulisporolides, SCH-47554/46555, and lobosamide A/B/C (Table S8), suggesting differences between strains. Therefore, the high number of BGCs and their variation in encoding metabolites in these strains suggest differences in their biosynthetic potential, thus indicating the unique secondary metabolite biosynthetic potential of each strain.

### Putative antifungal clusters in *S. chrestomyceticus* B5

**Fig 5:**
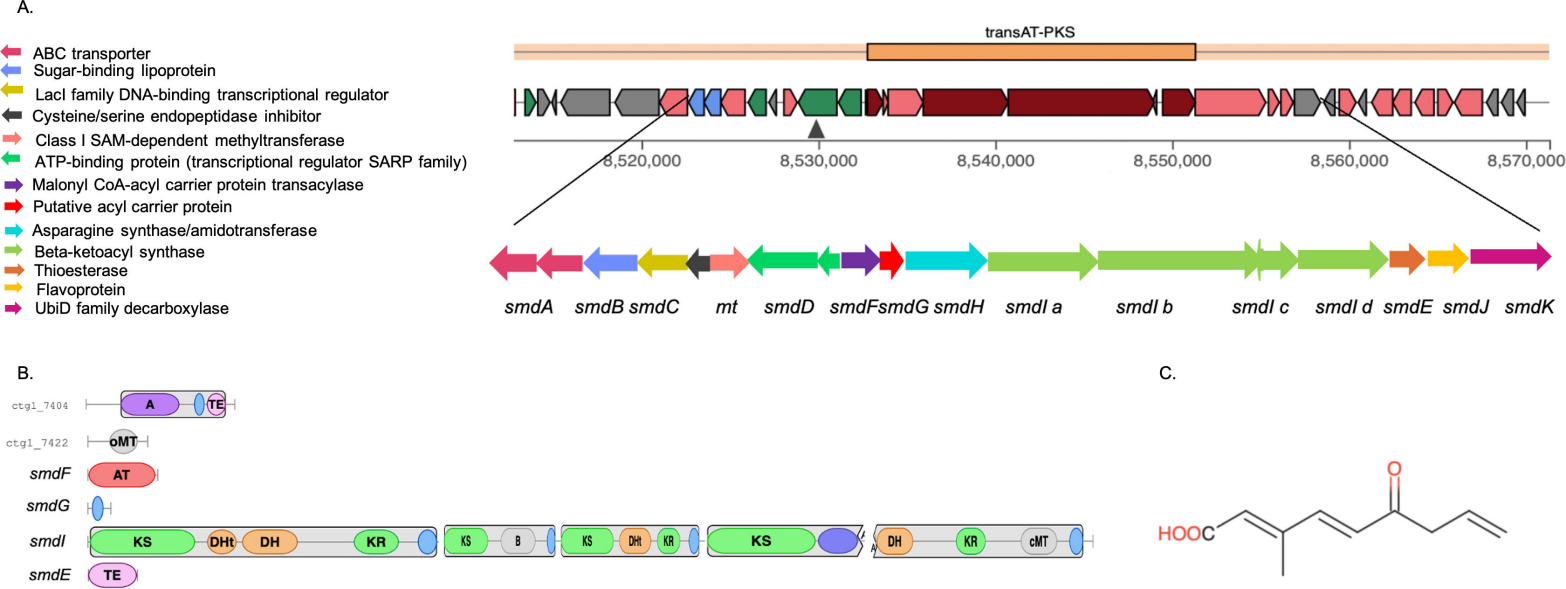
*In silico* analysis of putative transAT-PKS cluster with predicted structure linked to antifungal activity: (**A**) gene cluster organization with ORFs of the transAT-PKS biosynthetic pathway. (**B**) Domain architecture of the encoded PKS enzymes. Each module is represented by a box with individual domains labeled. (**C**) Predicted core chemical structure from the transAT-PKS cluster.

Since most of the BGC-encoded metabolites are reported to have antibacterial and antitumor activities. Surprisingly, the antimicrobial assay suggested that *S. chrestomyceticus* B5 possesses strong antifungal activity against different fungal pathogens. Therefore, BGCs were analyzed for antifungal clusters in its genome. A biosynthetic cluster showed 55% similarity with the bafilomycin B1 cluster. Bafilomycin has been reported to exhibit bioactivity against *A. fumigatus* (35). However, *S. chrestomyceticus* B5 also showed activity against *Candida* species, indicating the possible presence of additional or distinct bioactive metabolites. Therefore, BGCs with low similarity to known clusters were also examined. Among them, a transAT-PKS cluster with 57.9 kb size showed 27% and 19% similarity to cycloheximide and 9-methylstreptimidone clusters, respectively (Fig. S1). These compounds belong to polyketide glutarimide antibiotics, reported for antifungal activities (36, 37). This transAT-PKS cluster was conserved in all *S. chrestomyceticus* genomes; three BGCs were present together in a BGC region (aminopolycarboxylic-acid, transAT-PKS, and NAPAA clusters) in the other reference genomes, whereas in the *S. chrestomyceticus* B5 genome, two BGCs (NAPAA and transAT-PKS clusters) were present. In the transAT-PKS cluster of *S. chrestomyceticus* strains, variation in the gene organization was observed. The core biosynthetic gene was split into four ORFs in *S. chrestomyceticus* B5, whereas it was present as a single ORF in the other three strains (Fig. S1). This cluster comprises a modular PKS and tailoring genes. It contains a standalone acyltransferase (AT) gene, instead of an integrated AT domain within PKS modules, thus supporting transAT biosynthesis. The core megasynthetases consist of modules predicted to incorporate malonyl-CoA (mal), methylmalonyl-CoA (Me-mmal), and cyclohexyl-malonyl (ccmal) extender units, suggesting the assembly of a structurally diverse and functional polyketide. These modules harbor ketosynthase (KS), acyl carrier protein (ACP), ketoreductase (KR), dehydratase (DH), β-branching domain (B), and C-methyltransferase (cMT), which incorporates methyl group. A terminal thioesterase (TE) domain is present downstream of the modules, which is responsible for polyketide chain release. In addition to that, the cluster encodes tailoring enzymes, including alcohol dehydrogenase (AbH) and O-methyltransferase (oMT), which catalyze oxidation and methylation steps (Fig. 5). These features suggest that this BGC is responsible for the synthesis of a bioactive polyketide in strain *S. chrestomyceticus* B5. Given its intriguing architecture, the cluster was further analyzed and compared with the 9-methylstreptimidone biosynthetic cluster that could be responsible for the possible bioactivity against yeasts. Five core genes (*smdF, smdG, smdH, smdI,* and *smdE*) are required for the synthesis of 9-methylstreptimidone, as reported from previous literature (38) (Fig. S2). The gene-to-gene alignment between the transAT-PKS and 9-methylstreptimidone cluster suggested similarity in the *smdF*, *smdG, smdH*, *smdJ,* and *smdK* genes. However, there is no significant similarity present between the genes (*smdE* and *smdI*) of both pathways. The sequence homology also suggested similarity between genes of 9-methylstreptimidone (*smdH, smdI, smdJ,* and *smdK*) and the transAT-PKS cluster (Fig. 4C). However, the *smdI* gene in the transATPKS cluster was present in four ORFs in *S. chrestomyceticus* B5, and all of them showed more than 75% similarity with the *smdI* gene of the 9-methylstreptimidone cluster (Table S9), suggesting the possibility of some derivative of 9-methylstreptimidone based on sequence homology of the genes.

Therefore, based on genome mining, we hypothesized that this pathway might be responsible for the bioactivity against *Candida* species and might synthesize some derivative of these antifungal compounds. *S. chrestomyceticus* B5 was subjected to bioactivity-guided purification to identify the antifungal compound.

### Bioactivity assay and minimum inhibitory concentration of *S. chrestomyceticus* B5 crude extract

The fermented *S. chrestomyceticus* B5 supernatant was extracted using ethyl acetate to get a yellow crude extract, and it was estimated for bioactivity against bacteria and fungi. The bioactivity assay suggested that the crude extract possesses both antibacterial and antifungal activity against test pathogens. The extract showed activity against fungi *A. fumigatus, C. glabrata*, *C. albicans,* and against bacteria *S. aureus*. However, it showed weak activity against *E. coli,* as it formed a small zone of inhibition (Fig. S3). The results indicated that the extract exhibits a notable antibacterial effect against *S. aureus,* indicated by an MIC of 1 μg/mL, whereas MIC was not detected for *E. coli*. The crude extract was also potentially active against yeasts *C. glabrata* and *C. albicans*. The MICs were 5 μg/mL and 2 μg/mL, respectively, as well as 1 μg/mL for *A. fumigatus* (Table S10).

### Purification of antimicrobial compounds by chromatography

**Fig 6:**
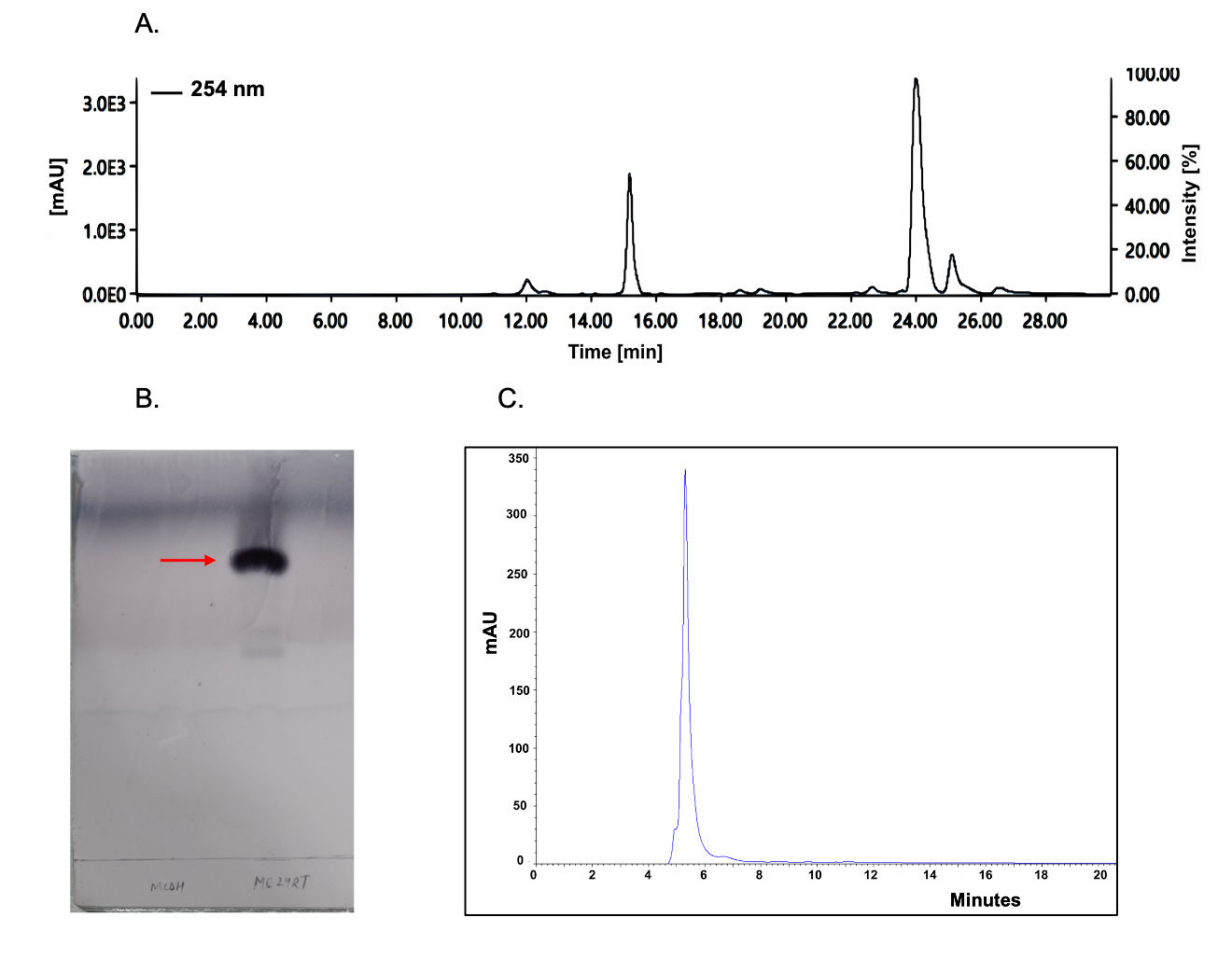
Purification of antifungal compound through HPLC and TLC: (**A**) high-performance liquid chromatography to purify bioactive fractions collected from column purification. The antifungal compound was identified at a retention time of 24 min with UV absorption at 254 nm, expressed in milli-Absorbance Units (mAU). (**B**) The purified antifungal compound was visualized on TLC using anisaldehyde-H_2_SO_4_ reagent. The bioactive compound was identified as a purple spot present at R_f_ 0.75. (**C**) The purity of the antifungal compound was checked on HPLC, confirming its purity with a single resolved peak.

The column-purified fractions were screened against *C. albicans*, *A. fumigatus, C. glabrata, E. coli*, and *S. aureus*. Two different bioactivities were observed: an antifungal activity against *C. glabrata* in ethyl acetate:methanol (8:2) separated fractions and an antibacterial activity against *S. aureus* in methanol:water (8:2) separated fractions. However, these fractions were not active against *E. coli*, *C. albicans,* and *A. fumigatus*. Furthermore, the bioactive fractions active against *C. glabrata* were separated using reverse-phase HPLC. The bioactive peak from HPLC separation was obtained at 24 min retention time (RT), detected at a wavelength of 254 nm (Fig. 6A). After HPLC purification, the bioactive antifungal compound was further purified by thin-layer chromatography to get a single purified spot of antifungal compound at R_f_ 0.75 (Fig. 6B) as a yellow solid, and it was soluble in methanol and purity was confirmed on HPLC as a single peak (Fig. 6C).

### Identification of the compound from LC-MS and FTIR analysis

The purified compound was analyzed on LC-MS, which exhibited an m/z of 294.1 [M+H]^+^ in positive ionization mode at the retention time of 8.6 min, and an m/z of 292.1 [M−H]^−^ in negative ionization mode at the same RT, which provided the molecular mass of the antifungal compound as 293.1 (Fig. S4 and S5). Furthermore, the functional groups present in the compound were confirmed from FTIR analysis. The peaks present in region 1259.14, 1403.08, 1436.41, 1452.72, 1566.58, and 1570.01 suggested C−N stretching and N−H deformation, whereas the peaks at 1606.64, 1609.12, 1613.17, 1695.69, 1701.55 suggested C=C−C aromatic ring stretch, N−H, and C=O stretching. The peak at 2355.73 suggested the presence of a −CH2 group (alkyl group), and the major wide peaks in the region of 2926.68, 2969.44, 3088.58, 3324.01, 3363.31, and 3548.62 suggested C−H stretching in aromatic ring and O−H stretching (Fig. S6).

**Fig 7:**
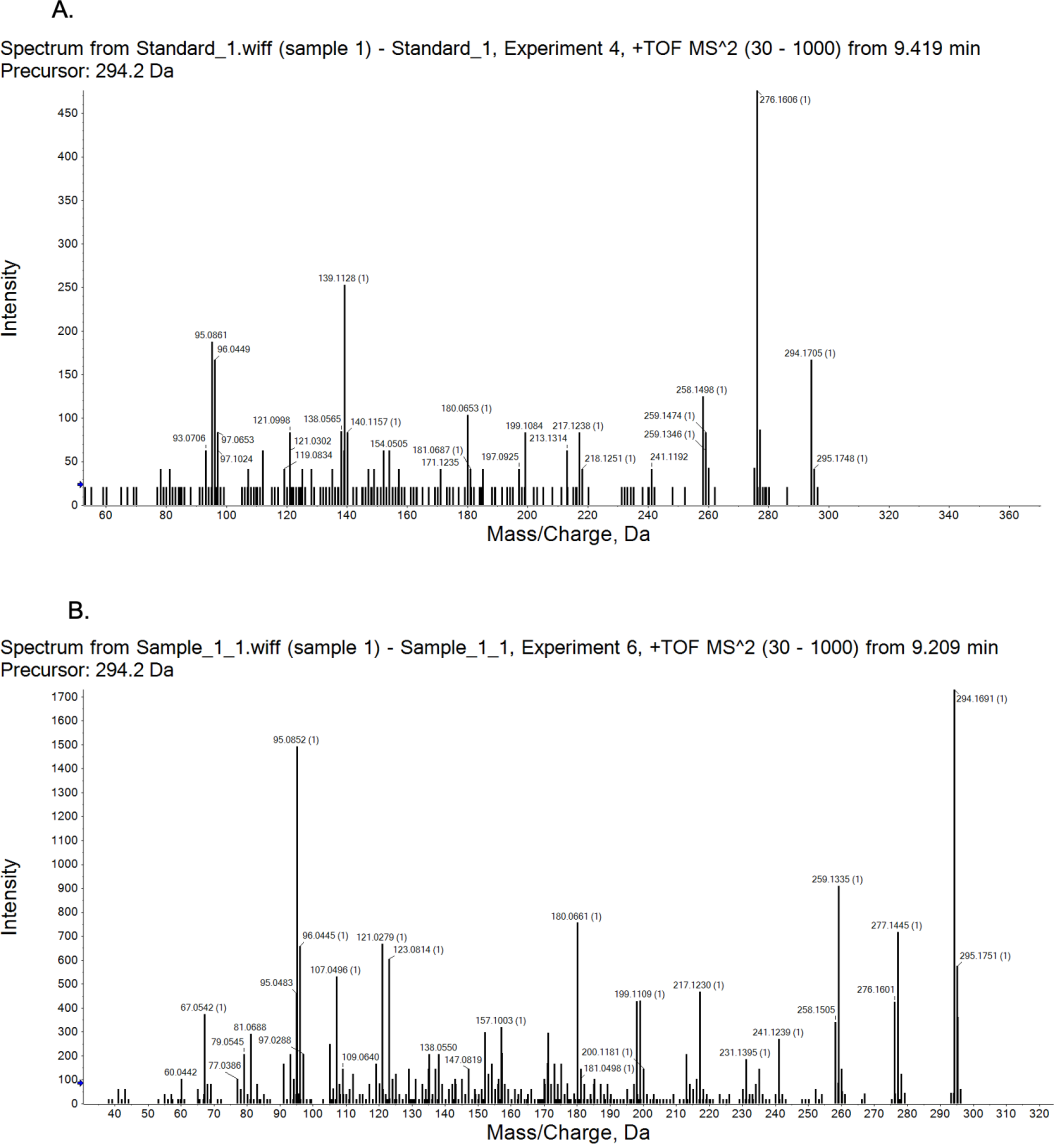
Confirmation of purified compound as streptimidone derivative or isomer: (**A**) MS/MS of standard streptimidone. (**B**) MS/MS of purified B5 antifungal compound.

For the structural confirmation of the compound, the fragmentation pattern of the proposed compound was matched with that of the standard streptimidone (Cayman Chemicals). This resulted in confirmation of the purified compound as a streptimidone derivative or isomer since the fragmentation spectra of the purified compound matched with the fragmented ion spectra of streptimidone in positive ion scan mode (fragmented peaks were at m/z 198.07, 276.16, 180.06, 156.06, 138.05, 95.08, 67.05, and 60.04) (Fig. 7).

**Fig 8:**
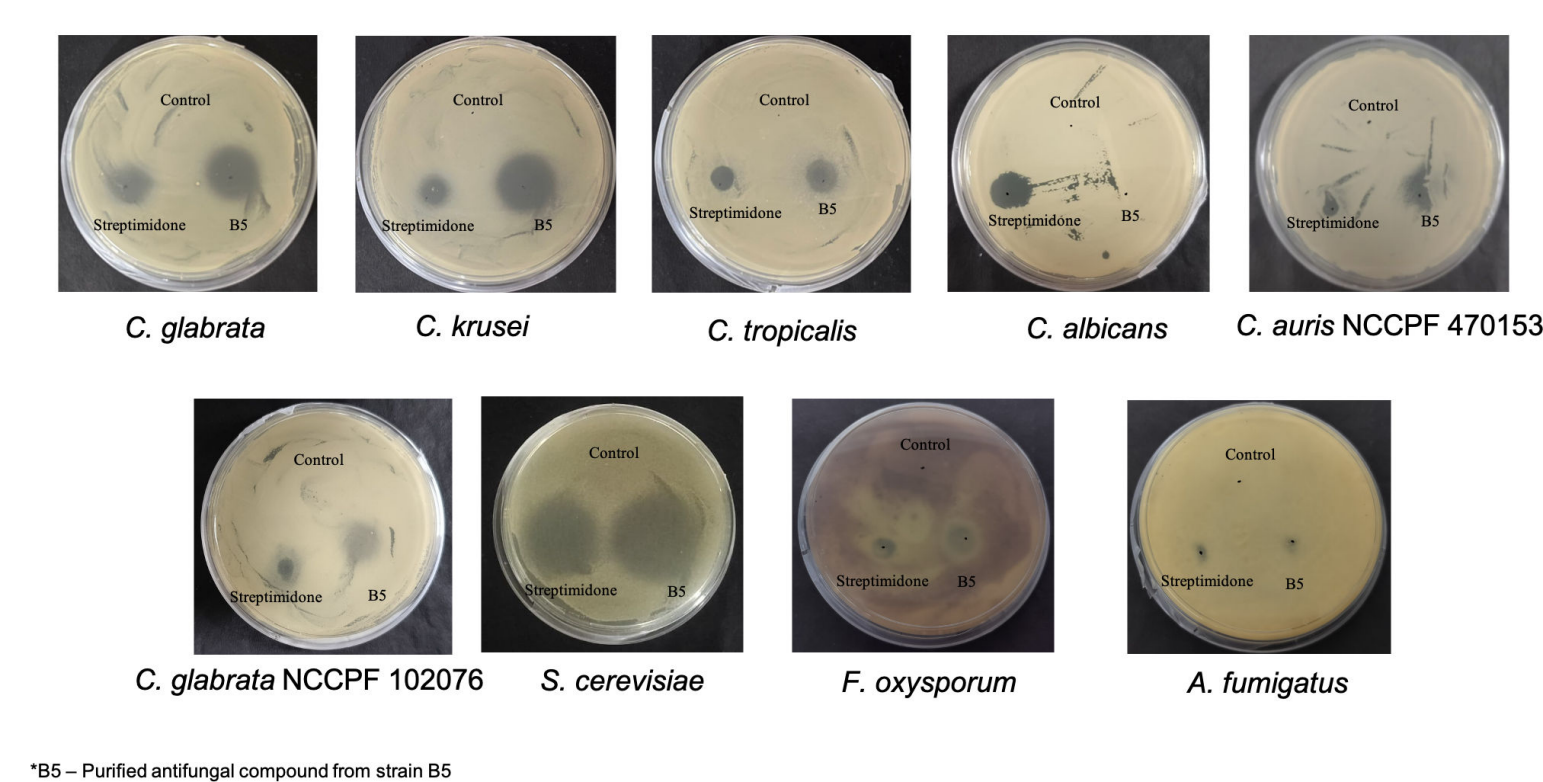
Spot-on-lawn assay of purified streptimidone derivative or isomer against fungal pathogens: the purified compound and synthetic streptimidone were dissolved in AMQ, and 5 µL of the streptimidone and the purified compound was put on culture plates of different fungal pathogens. The streptimidone and purified compound showed a similar pattern of bioactivity against *C. glabrata*, *C. krusei*, *C. tropicalis*, *S. cerevisiae*, *F. oxysporum,* drug-resistant *C. auris* and *drug-resistancet C. glabrata,* except *C. albicans*.

The synthetic streptimidone and purified compound were assayed for bioactivity against fungal pathogens, showing a similar pattern of bioactivity against different species of *Candida and F. oxysporum,* except *C. albicans*. The streptimidone and purified compound were also bioactive against *C. glabrata* and drug-resistant *C. auris* (Fig. 8; Fig. S7).

## DISCUSSION

Infectious diseases caused by bacterial and fungal pathogens are emerging as a major global health threat, and the rise of drug resistance in these pathogens would cause further complications in treating infections caused by them (39, 40). Therefore, finding bacteria with potential antimicrobial activities can be one way to find molecules that can be effective against them. *Streptomyces* are major producers of antimicrobial compounds with known greater potential (11). In this study, a *Streptomyces* species isolated from garden soil possesses wide antimicrobial activity against bacterial and fungal pathogens. Previous studies have isolated *Streptomyces* species from garden soil, which reported antimicrobial activities against *E. coli*, *S. aureus*, *Bacillus* species, *Candida* species, *Fusarium,* and multidrug-resistant bacteria (31). A new antibiotic, lariocidin, with broad-spectrum activity against different bacterial pathogens, has been isolated from a garden soil bacterium (41). Our isolate was also potentially active against these pathogens, suggesting garden soil contains *Streptomyces* species that can provide a diverse range of antimicrobial metabolites, possibly due to the competitive soil environment present in garden soil.

The strain B5 was identified as *S. chrestomyceticus*. The genome annotation with other reference strains suggested that the strains contain a high number of genes in metabolism, especially carbohydrates and amino acids. The primary source of energy for any soil bacteria is amino acids and carbohydrates (42). Therefore, the carbohydrates and amino acids might act as the source of carbon and nitrogen and might be helpful in the utilization of nutrients released from plant biomass (43, 44). *S. chrestomyceticus* B5 possesses genes for stress response, including DNA repair pathways, oxidative stress, osmotic stress, and heavy metal resistance genes, suggesting a role in the protection of bacteria from such stressors. Previously, a study has identified heavy metals including Pb, Mn, Ni, Zn, Fe, Cd, and Cr in soil from the national capital region (45). Another study has documented heavy metal contamination in the groundwater of the Delhi region (46). The exposure to heavy metal stress results in the production of reactive oxygen species and may also result in DNA damage (47). Besides, plants also produce reactive oxygen species as byproducts in soil, which can be harmful to the bacteria living in soil (48). Therefore, these implications might indicate stress response by soil bacteria. The genome annotation suggested that *S. chrestomyceticus* B5 possesses PGP traits, including genes for auxin biosynthesis, siderophore production, nitrite and nitrate ammonification, denitrification, and phosphate solubilization. These traits suggest that *S. chrestomyceticus* B5 might be in a symbiotic relationship with plants, thus might be helpful for plant growth (49, 50).

Since *S. chrestomyceticus* B5 possesses wide antimicrobial activity, the genome mining approach was employed to compare the BGCs with other reference genomes of this strain. The core BGCs present across all strains were desferroxamine B, hopene, griseobactin, geosmin, ectoine, terpenes, and isorenieratene. These essential metabolites are mostly useful for *Streptomyces* present in soil due to their role in iron acquisition from soil, mitigation of osmotic stress, stabilizing the plasma membrane, and protection of microbial cells from oxidative stress (51–54). Besides these metabolites, these strains also possess BGCs encoding various antibacterial and antitumor metabolites. All the strains contain BGC for paromomycin, suggesting this BGC is conserved in all strains of *S. chrestomyceticus*. Paromomycin is an aminoglycoside antibiotic reported for broad-spectrum activity, targeting both gram-negative and gram-positive bacterial species (55). The antibacterial effect against *E. coli* might be due to the paromomycin cluster, since paromomycin is reported to inhibit *E. coli* (56).

Another cluster that showed similarity to the bafilomycin B1 cluster, possibly encoding a similar compound, might be responsible for bioactivity against *A. fumigatus*, as this compound is also reported for inhibiting filamentous fungi (35). Likewise, these bacteria also possess BGCs that could be involved in the synthesis of other bioactive molecules. However, in-depth BGC analysis indicated that different metabolites are produced by each strain, thus indicating differential biosynthetic diversity in these strains. They could be involved in the biosynthesis of some novel molecules, as these strains exhibited very low similarity to the known BGC clusters. Previous studies have also reported strain-level diversity in *Streptomyces* species (57, 58).

A BGC with low homology to an antifungal cluster was observed in all strains, identified as a transAT-PKS cluster, and showed very low similarity to cycloheximide and 9-methylstreptimidone clusters. These compounds belong to glutarimide-containing polyketides, comprising over 50 bioactive molecules with diverse activities that have been isolated from *Streptomyces* species (59). Cycloheximide and 9-methylstreptimidone are inhibitors of protein biosynthesis targeting the 60S subunit of the ribosome, thus inhibiting various phytopathogenic fungi (60). The 9-methylstreptimidone is one of the derivatives of the streptimidone compound reported from *Streptomyces* species; it contains a terminal alkene and possesses antifungal activities (61, 62). The cluster for biosynthesis of streptimidone is not known; however, a previous study has reported a putative BGC for streptimidone based on the similarity of the cluster with the 9-methylstreptimidone cluster. The biosynthesis of these compounds proceeds by extension of polyketide using trans-acyltransferase, which contains the domain for the synthesis of glutarimide ring and the incorporation of other −CH_3_ substituents (38, 62).

This transAT-PKS cluster predicted through genome mining, which showed low homology to the 9-methylstreptimidone cluster, could be involved in the biosynthesis of streptimidone derivative or isomer, which consists of four core polyketide genes, along with two methyltransferases, putative acyl carrier, amidotransferase, malonyl CoA-acyl carrier protein transacylase, putative thioesterase, flavoprotein, and decarboxylase, which might be required for biosynthesis of streptimidone scaffold, similar to the genes for biosynthesis of 9-methylstreptimidone (38) (Fig. 5). Other than the required genes in 9-methylstreptimidone, the transAT-PKS cluster’s core gene was present in multiple ORFs in *S. chrestomyceticus* B5. This cluster also contains additional genes, which may further enhance the structure of the existing molecule and might give rise to some novel compound. Therefore, the genome mining approach can be used to guide and identify the putative bioactive compounds.

The *S. chrestomyceticus* B5 crude extract exerted antibacterial and antifungal activities against *S. aureus*, *C. glabrata*, *C. albicans,* and *A. fumigatus*. With bioactivity-guided fractionation, an antifungal fraction active against *C. glabrata* and another fraction active against *S. aureus* were separated. However, these fractions showed no activity against *A. fumigatus, C. albicans,* and *E. coli*, suggesting five different bioactive molecules are produced by *S. chrestomyceticus* B5 that could be responsible for these different bioactivities observed in this strain. The bioassay-guided purification of the antifungal fraction active against *C. glabrata* resulted in the identification of a streptimidone derivative or isomer, which exhibited fragmented peaks similar to those reported in an earlier study (63). The purified antifungal compound (streptimidone derivative or isomer) and synthetic streptimidone inhibited *F. oxysporum* and *Candida* species, including *S. cerevisiae* and *C. glabrata*; however, the purified compound did not inhibit the growth of *C. albicans*. Previous studies have also reported that the naturally isolated streptimidone exhibits activity against yeasts; however, it is ineffective against *C. albicans* even up to a concentration of 50 μg/mL (61, 62). The synthetic streptimidone exerted bioactivity against *C. albicans,* possibly because the streptimidone stereoisomers are known to exert differential bioactivity against *S. cerevisiae* (64); this different bioactivity could be due to a different stereoisomer of synthetic streptimidone. The purified antifungal compound was also active against drug-resistant *C. auris*, which is a multidrug-resistant pathogen responsible for bloodstream infections and associated with 60% mortalities (4).

Therefore, screening of pre-existing antifungal compounds can be one of the effective ways of fighting against these leading pathogens. Genome mining can predict the known molecules based on the similarity to known clusters, but the prediction of BGCs with minimal similarity to known reference clusters does not rule out the possibility of encoding the known compound, since the streptimidone pathway does not exist in the MIBiG database. Due to this limitation, the possible compound was not predicted from genome mining. This study highlighted the antimicrobial potential of *S. chrestomyceticus* through bioactivity assay and also its possession of multiple biosynthetic pathways, with unknown clusters as well, suggesting the possibility of encoding other bioactive metabolites, which could be responsible for other observed bioactivities.

### Conclusion

*S. chrestomyceticus* B5 holds potential against a wide range of pathogens with antibacterial and antifungal activities. It contains plant growth-promoting traits, stress response and DNA repair genes, and NRPS and PKS pathways with antibacterial and antitumor activities, along with two putative antifungal clusters. The crude extract exerted strong antimicrobial activities, and bioassay-guided purification identified an antifungal compound active against *Candida* species and drug-resistant *C. auris*. *S. chrestomyceticus* B5 also possesses different bioactivities against other potential pathogens; therefore, identifying such antimicrobial compounds using genome mining approaches and bioassay-guided purification can help in the fight against antimicrobial-resistant pathogens.

## Data Availability

The genome sequence of the strain in this study has been deposited in the National Center for Biotechnology Information (NCBI) under the bioproject accession number PRJNA1295772.

## SUPPLEMENTAL MATERIAL

Supplemental figures spectrum.02688-25-s0001.pdf Fig. S1 to S7.

Supplemental tables spectrum.02688-25-s0002.xlsx Tables S1 to S10.

ASM does not own the copyrights to Supplemental Material that may be linked to, or accessed through, an article. The authors have granted ASM a non-exclusive, world-wide license to publish the Supplemental Material files. Please contact the corresponding author directly for reuse.
