# Supplementary material for: Genomic insights and biosynthetic gene cluster analysis of Streptomyces chrestomyceticus strain B5 showing antimicrobial activities
Source: Microbiol Spectr. 2026 Mar 30;14(5):e02688-25. doi: 10.1128/spectrum.02688-25 (PMC13141997; doi:10.1128/spectrum.02688-25)
Supplement: Supplemental figures — Fig. S1 to S7. [file spectrum.02688-25-s0001.pdf]

Supplementary Figures

Genomic insights and biosynthetic gene cluster analysis of *Streptomyces chrestomyceticus* B5 strain showing antimicrobial activities.

Aruna Kumari[1,2], Sonam Nain[1,2#], Praveen Singh[1,2$], Nar Singh Chauhan[1&], Swarnendu Bag[1,2], Rakesh Sharma[1,2*]

*Correspondence: Rakesh Sharma (rakesh.igib@csir.res.in)

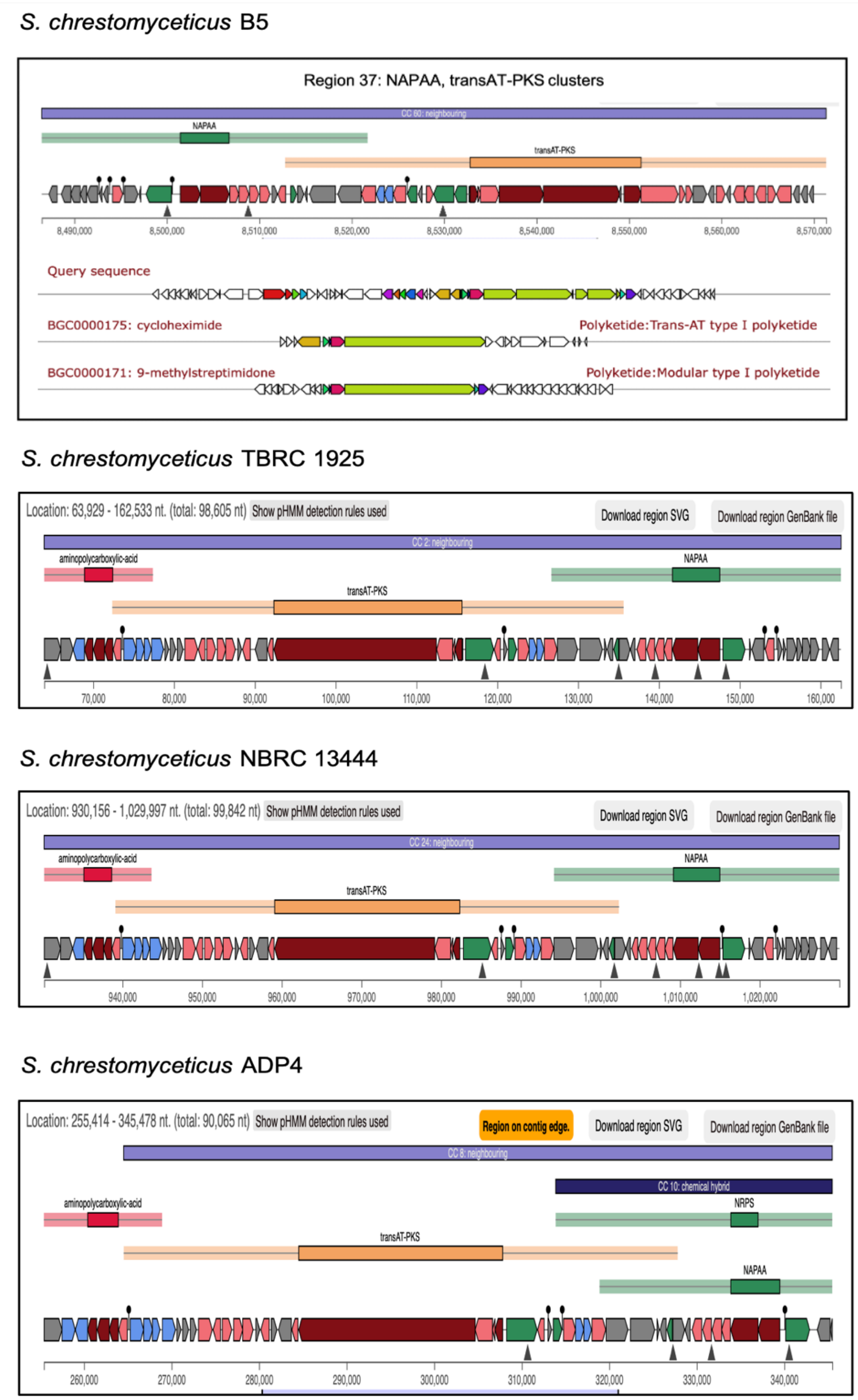

Figure S1: A transAT-PKS cluster identified as a putative antifungal cluster and conserved in all strains: The *S. chrestomyceticus* B5 genome contains two BGCs corresponding to NAPAA and the transAT-PKS cluster. The transAT-PKS cluster showed 27%

similarity with the cycloheximide and 19% similarity with the 9-methylstreptimidone gene clusters. This cluster was identified in all strains; however, the core gene was present in multiple ORFs in *S. chrestomyceticus* B5, while in other strains, it was present as a single ORF.

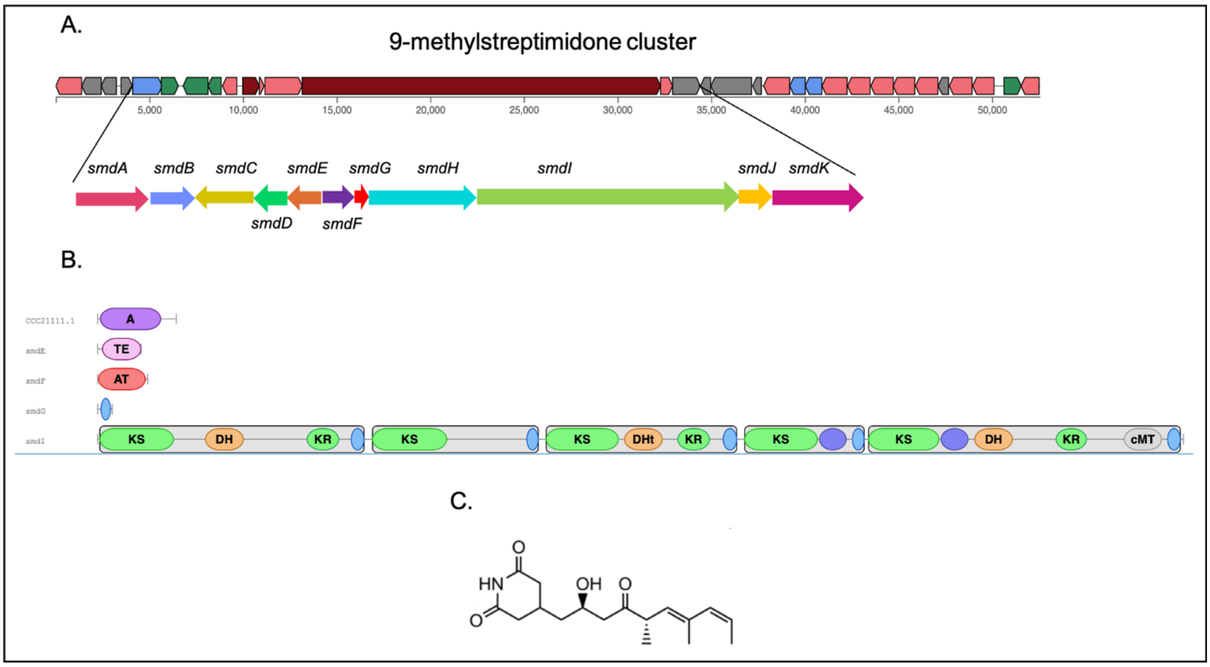

Figure S2: 9-methylstreptimidone biosynthetic cluster from *Streptomyces himastatinicus* ATCC 53653. (A) Gene cluster map of 9-methylstreptimidone from the MIBiG database. (B) PKS domains of 9-methylstreptimidone. (C) Chemical structure of 9-methylstreptimidone.

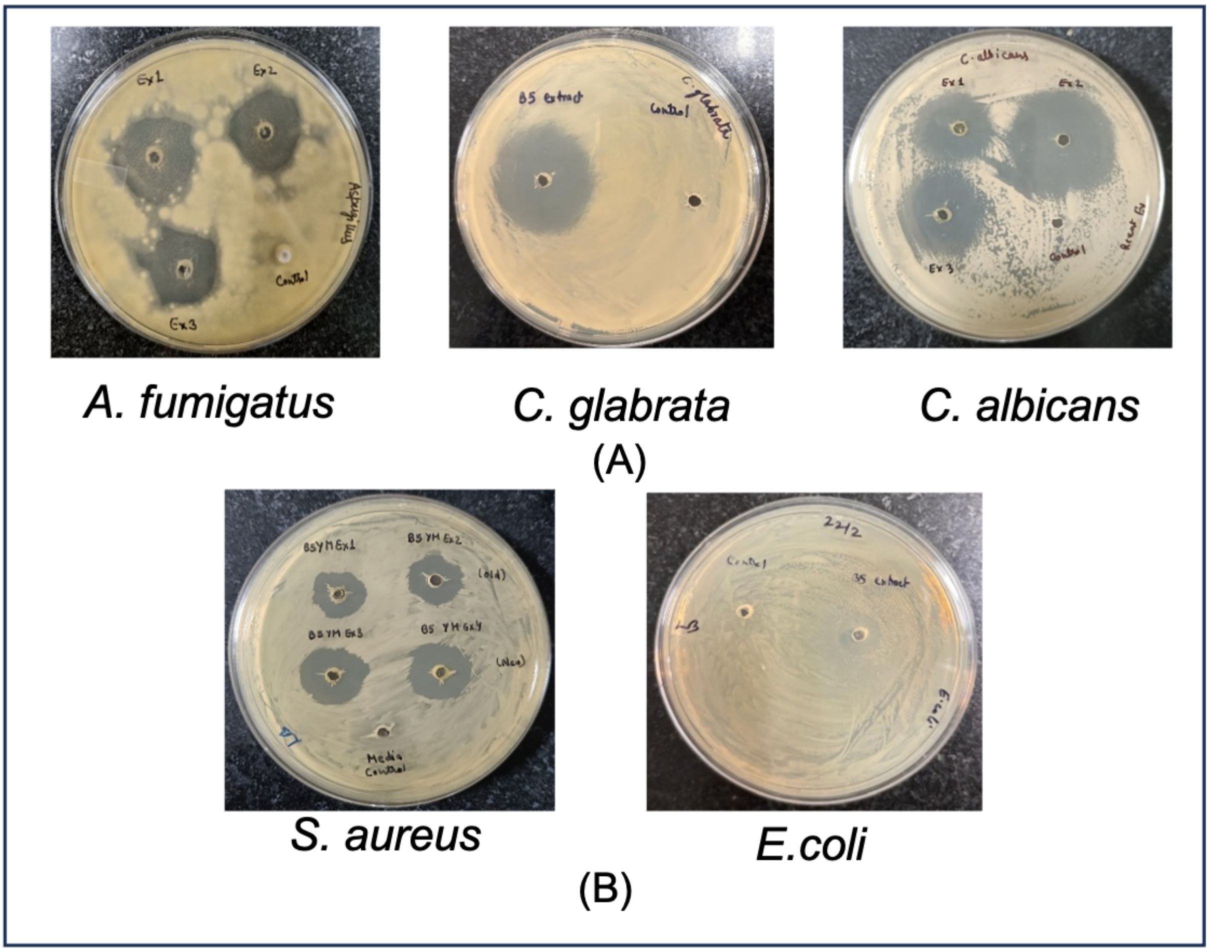

Figure S3: Bioactivity assay of *S. chrestomyceticus* B5 crude extract against test pathogens using agar well diffusion method: (A) The *S. chrestomyceticus* B5 crude extract displayed bioactivity against fungi *A. fumigatus*, *C. glabrata* and *C. albicans.* The crude extract was also active against gram-positive *S. aureus*; however, it was least active against gram-negative *E. coli*.

A.

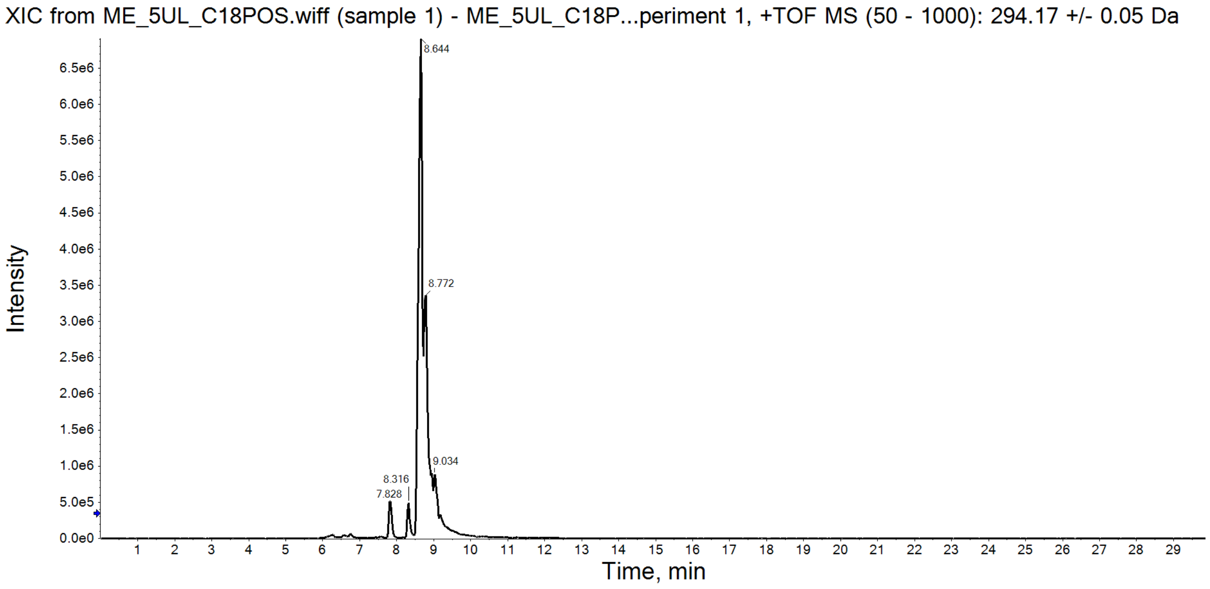

B.

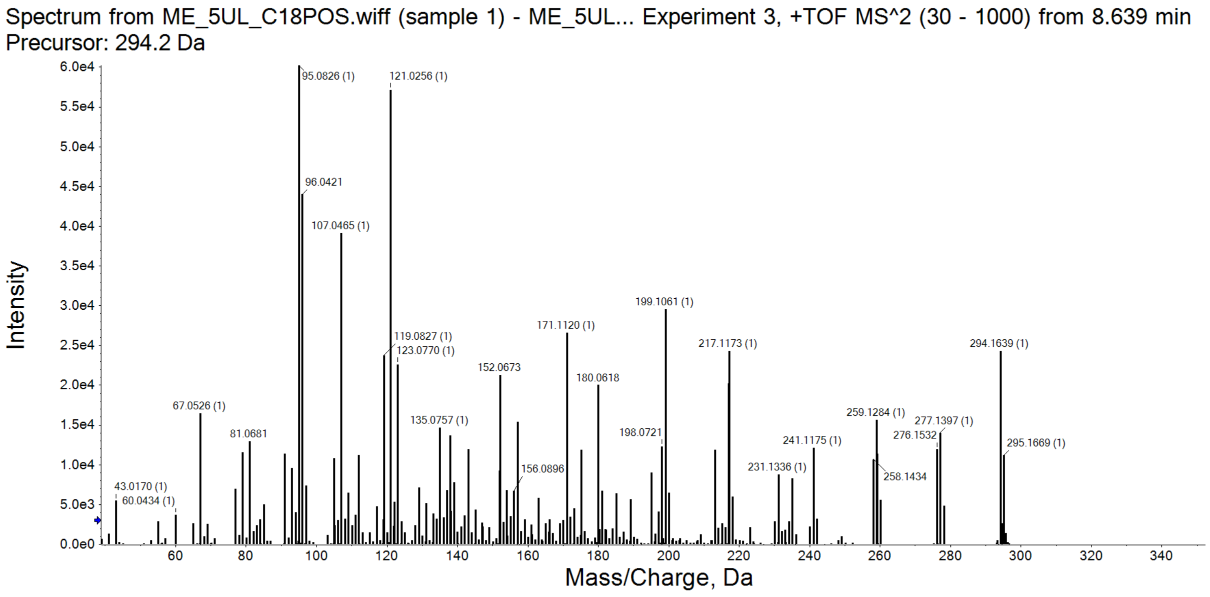

Figure S4: Identification of antifungal compound through mass spectrometry in positive ionization mode: (A) Extracted ion chromatogram of purified antifungal compound obtained at RT 8.6 min in positive ionization mode. (B) Fragmentation spectra of m/z 294.1 in positive ionization mode.

A.

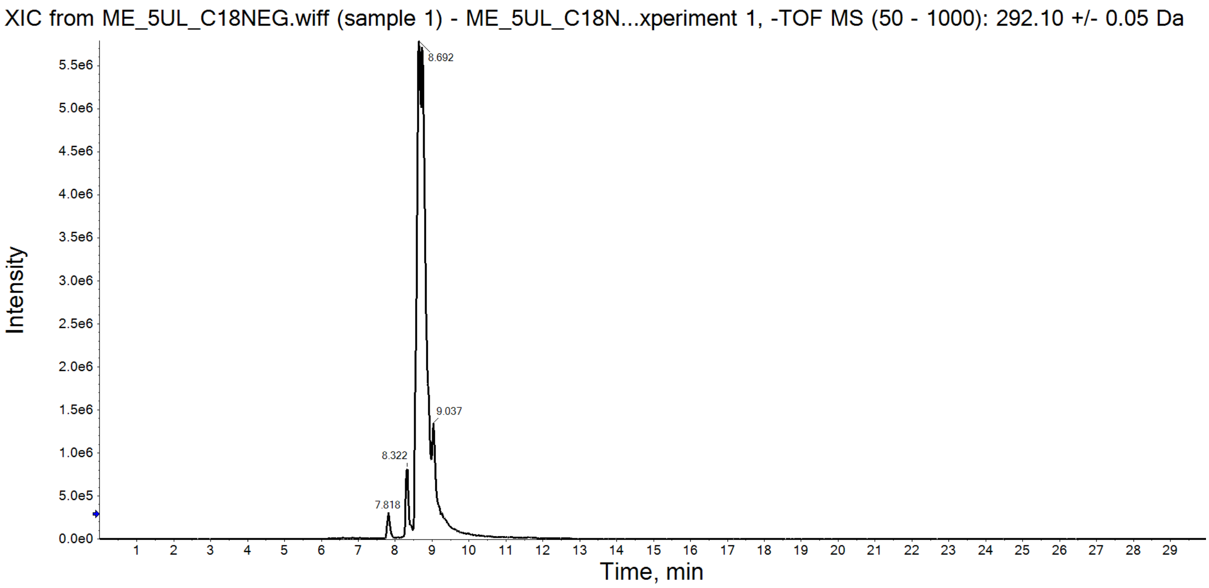

B.

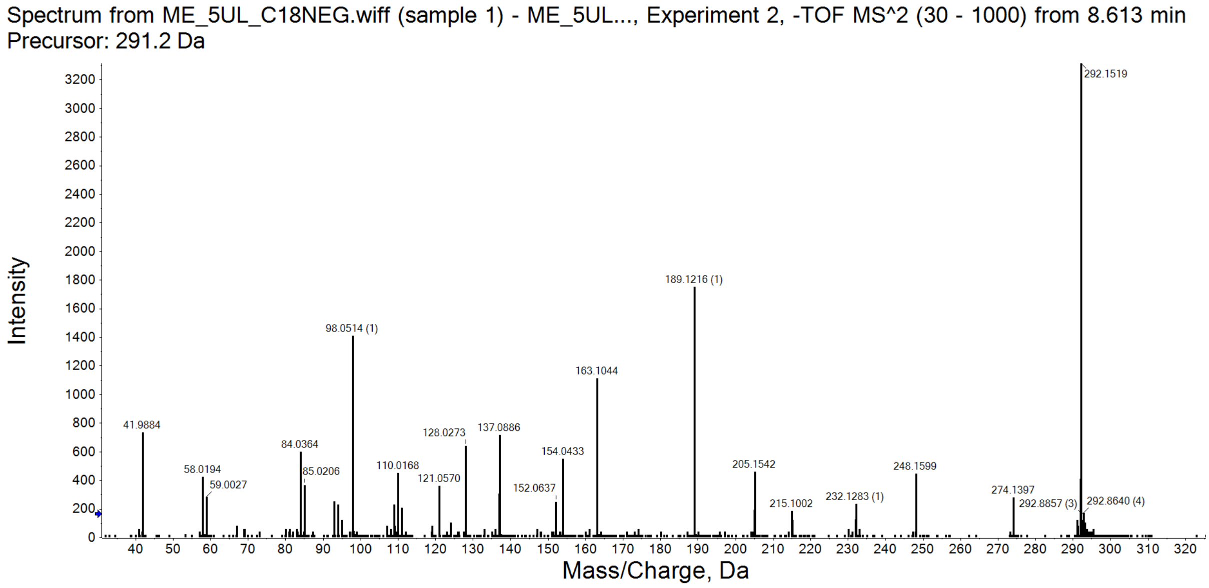

Figure S5: Identification of antifungal compound through mass spectrometry in negative ionization mode: (A) Extracted ion chromatogram of purified antifungal compound obtained at RT 8.6 min in negative ionization mode. (B) Fragmentation spectra of m/z 292.1 in negative ionization mode. Thus, confirming the mass of the antifungal compound as 293.1.

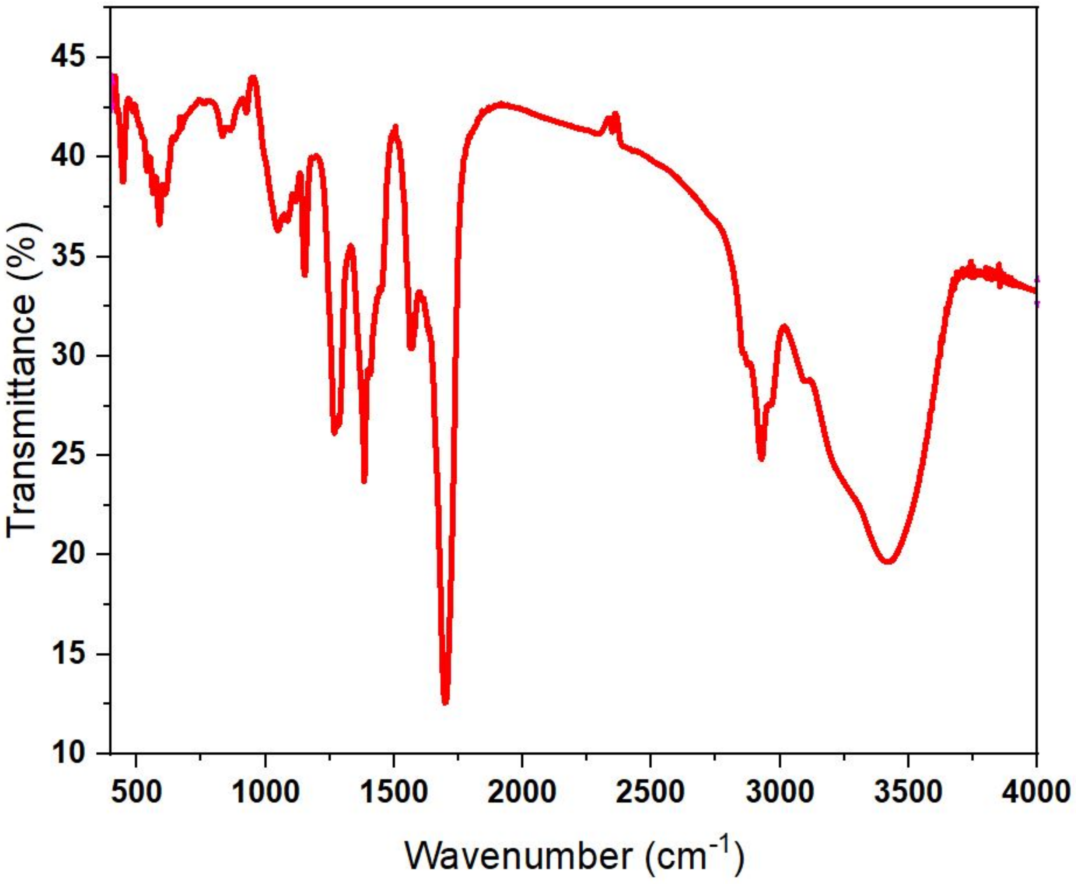

Figure S6: Infrared spectrometry of purified antifungal compound: The functional groups present in the purified antifungal compound were identified by infrared spectroscopy.

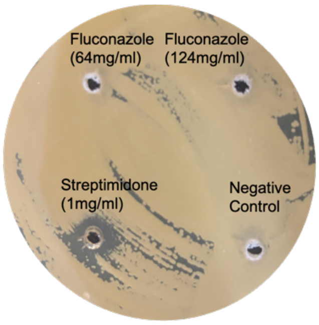

Figure S7: Agar well diffusion of standard streptimidone against *C. auris*: Bioactivity assay of streptimidone (1mg/ml) against drug-resistant *C. auris*.
